# Modeling the Tumor Microenvironment of Ovarian Cancer: The Application of Self-Assembling Biomaterials

**DOI:** 10.3390/cancers13225745

**Published:** 2021-11-16

**Authors:** Ana Karen Mendoza-Martinez, Daniela Loessner, Alvaro Mata, Helena S. Azevedo

**Affiliations:** 1 School of Engineering and Materials Science, Queen Mary University of London, Mile End Road, London E1 4NS, UK; a.mendozamartinez@qmul.ac.uk; 2 Institute of Bioengineering, Queen Mary University of London, Mile End Road, London E1 4NS, UK; 3 Department of Chemical Engineering, Faculty of Engineering, Monash University, Melbourne, VIC 3800, Australia; daniela.loessner@monash.edu; 4 Department of Materials Science and Engineering, Faculty of Engineering, Monash University, Melbourne, VIC 3800, Australia; 5 Department of Anatomy and Developmental Biology, Biomedicine Discovery Institute, Faculty of Medicine, Nursing and Health Sciences, Monash University, Melbourne, VIC 3800, Australia; 6 Max Bergmann Center of Biomaterials Dresden, Leibniz Institute of Polymer Research Dresden e.V., 01069 Dresden, Germany; 7 School of Pharmacy, University of Nottingham, Nottingham NG7 2RD, UK; a.mata@nottingham.ac.uk; 8 Department of Chemical and Environmental Engineering, University of Nottingham, Nottingham NG7 2RD, UK; 9 Biodiscovery Institute, University of Nottingham, Nottingham NG7 2RD, UK

**Keywords:** ovarian cancer, tumor microenvironment, peptides, biomaterial, self-assembly, mechanical properties, extracellular matrix, 3D models

## Abstract

**Simple Summary:**

The tumor-surrounding niche comprises not only cancer cells but also stromal cells, signaling molecules, secreted factors and the extracellular matrix. This niche has a three-dimensional (3D) architecture and is implicated in tumor progression, metastasis and drug resistance. 3D cancer models have been increasingly attracting attention due to their potential to provide a more representative tumor niche compared to traditional two-dimensional (2D) models. Bioengineered 3D models contain multiple cell types and important molecules that interact with each other to resemble crucial features of tumor tissues, including the 3D architecture, mechanical properties, genetic profile and cell responses to therapeutics. These defined characteristics highlight the application of 3D models to study tumor biology, metastatic pathways and drug resistance.

**Abstract:**

Ovarian cancer (OvCa) is one of the leading causes of gynecologic malignancies. Despite treatment with surgery and chemotherapy, OvCa disseminates and recurs frequently, reducing the survival rate for patients. There is an urgent need to develop more effective treatment options for women diagnosed with OvCa. The tumor microenvironment (TME) is a key driver of disease progression, metastasis and resistance to treatment. For this reason, 3D models have been designed to represent this specific niche and allow more realistic cell behaviors compared to conventional 2D approaches. In particular, self-assembling peptides represent a promising biomaterial platform to study tumor biology. They form nanofiber networks that resemble the architecture of the extracellular matrix and can be designed to display mechanical properties and biochemical motifs representative of the TME. In this review, we highlight the properties and benefits of emerging 3D platforms used to model the ovarian TME. We also outline the challenges associated with using these 3D systems and provide suggestions for future studies and developments. We conclude that our understanding of OvCa and advances in materials science will progress the engineering of novel 3D approaches, which will enable the development of more effective therapies.

## 1. Introduction

Ovarian cancer (OvCa) is one of the leading causes of cancer-related deaths among women, largely due to its late diagnosis, high metastatic potential and resistance to chemotherapy [1,2]. Due to the lack of specific clinical symptoms and early diagnosis, most patients are diagnosed at an advanced stage (FIGO stages III and IV) with intra-abdominal metastasis and the formation of ascites [2,3,4].

Distant metastasis occurs due to the shedding of cancer cells from the primary tumor, as spheroids or single cells, into the peritoneal cavity and the formation of ascites. Ascites, or tumor fluid, contains cellular components, cytokines, growth factors and other secreted molecules that support tumor cell proliferation and migration. This rich tumor-promoting microenvironment supports cancer cells to overcome apoptosis and inhibits the response to chemotherapy [5]. Tumor cells or spheroids in the ascitic microenvironment settle onto the mesothelial lining of the peritoneum, disaggregate and invade into the extracellular matrix (ECM) to form metastatic lesions [6,7].

In order to better understand the progression of OvCa, and to develop new and more effective therapeutic strategies, animal and 3D in vitro approaches have been engineered to recapitulate the unique TME of OvCa. Modeling OvCa is immensely complex due to diverse cell populations, pathological and genetic complexity (heterogeneity) and unknown recurrence mechanisms. If designed and implemented successfully, these approaches have the potential to improve cancer diagnosis and subsequent treatment.

In this review, we provide an overview of the key components of the ovarian TME. We also discuss selected examples designed to study the tumorigenesis of OvCa. In particular, we focus on self-assembly strategies that have been engineered to overcome the challenges presented by animal models and current 3D in vitro models. Readers are referred to the following reviews for complementary information [8,9,10].

## 2. Components of the Ovarian TME

**Figure 1:**
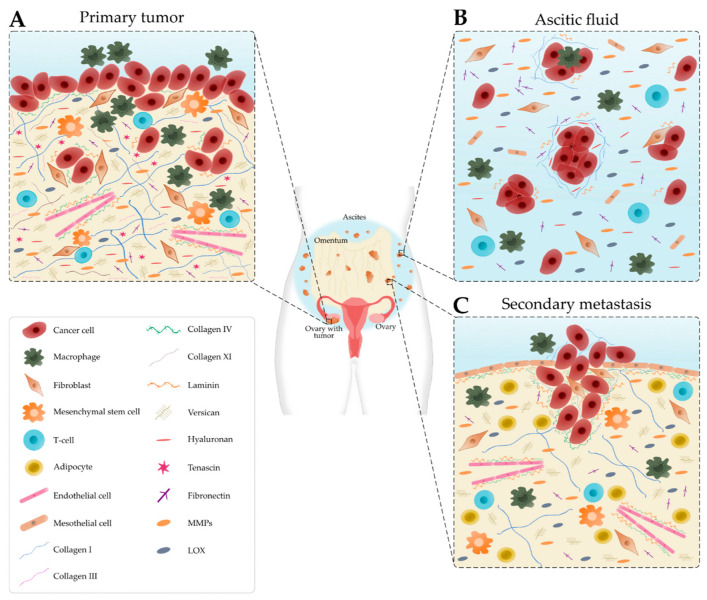
Schematic representation of the main cellular components and extracellular matrix composition of the ovarian tumor microenvironment depending on the disease site. (**A**) In the primary tumor, cancer cells recruit tumor-associated macrophages, cancer-associated fibroblasts, T-cells and endothelial cells. Many extracellular matrix components, such as fibronectin, hyaluronan, tenascin, versican, matrix metalloproteinases (MMPs) and lysyl oxidase (LOX) are upregulated. Collagen progressively remodels into thick fibrils and is randomly oriented. Laminin and collagen IV are underexpressed. (**B**) Detached single cells or spheroids are immersed in the ascitic fluid, which contains macrophages, fibroblasts, mesothelial cells and immune cells. Extracellular matrix components are found within the aggregated cells and the ascitic fluid. (**C**) Cancer cells settle onto the mesothelial lining to form secondary tumors that are rich in collagen. Laminin and collagen IV are overexpressed to promote metastasis. Levels of collagen I and III start to decrease.

The TME is comprised of cancer and stromal cells, signaling molecules, exosomes, and the ECM, and has long been implicated in the progression, metastasis and resistance to treatment [11,12]. Cellular and acellular components vary depending on the disease site, for example, the primary tumor site, ascites and secondary tumor site, which modulate the signals received by the cancer cells as summarized in Figure 1 [13].

### 2.1. Cellular Composition

OvCa cells are a diverse mix of cells with distinct properties and functions. Cancer stem cells (CSCs) represent a small sub-population that have properties of self-renewal, multi-lineage differentiation and resistance to anoikis [14]. CSCs contribute to tumor initiation, metastasis and resistance to treatment [15,16]. Several mechanisms within CSCs confer a survival advantage during treatment, including increased resistance to apoptosis, dormancy, the expression of ATP-binding cassette (ABC) transporters, the upregulation of aldehyde dehydrogenases (ALDHs), the response to DNA damage and epithelial-to-mesenchymal transition (EMT). As a result, CSCs lead to disease relapse by escaping treatment and repopulating the tumor with a heterogeneous and more aggressive population of cancer cells [17].

Within the stromal microenvironment, tumor-associated macrophages (TAMs) constitute the main population of immune cells in the primary tumor and ascites [18]. TAMs are not only key players in the implantation of cancer cells in the omentum, but also facilitate angiogenesis, metastasis and chemoresistance [18,19,20]. TAMs, together with myeloid-derived suppressor cells (MDSCs), contribute to the immune escape of cancer cells by hindering the cytotoxic activity of natural killer cells and cytotoxic T cells, inhibiting the maturation of dendritic cells (DCs) and recruiting regulatory T cells [18,21,22,23]. On the other hand, OvCa-derived exosomes can induce the polarization of macrophages in an M2-like phenotype and the apoptosis of DCs and lymphocytes [24].

OvCa cells reprogram stromal cells into a pro-tumoral phenotype, like for example, cancer-associated fibroblasts (CAFs). CAFs are reprogrammed fibroblasts that are crucial in the deposition and remodeling of the ECM through the secretion of MMPs and other secreted factors [25,26]. CAFs secrete pro-inflammatory cytokines (e.g., COX-2, CXL1, CCL5, CXC11 and IL-6) that induce EMT, cancer cell proliferation, invasion, chemoresistance and inhibit cancer cell apoptosis [25,27,28].

Mesenchymal stem cells (MSCs) can be converted to cancer-associated MSCs (CA-MSCs) and later differentiate into fibroblasts, osteocytes or adipocytes [29]. The upregulation of TGF-β in CA-MSCs increases the number of CSCs and chemoresistance [30,31]. Particularly, adipocytes located in the omentum attract cancer cells through the secretion of IL-8 and support cancer cell proliferation through fatty acids [32,33]. Adipocytes also secrete factors that enhance chemoresistance by activating the Akt pathway [33]. Elevated levels of VEGF secreted by tumor cells, TAMs, CAFs and adipocytes lead to angiogenesis, endothelial cell survival, proliferation, migration and vascular permeability [34].

### 2.2. Matrix Composition

One major constituent of the ovarian TME is the ECM, a meshwork of proteins (e.g., collagen, fibronectin, tenascin and laminin), glycosaminoglycans (e.g., hyaluronan), proteoglycans (e.g., versican) and remodeling enzymes (e.g., MMP2, MMP9 and LOX). The ECM provides both biomechanical and biochemical support for cancer cell proliferation and metastasis [10]. Continuous remodeling of the ECM, induced by cancer and stromal cells, changes its mechanical properties, thereby influencing disease progression and chemoresistance [35]. Additionally, cancer cells experience numerous mechanical stimuli within the ovarian TME, such as shear stress, compressive stress, tensile stress and stress relaxation, which directly affect their behavior and signaling pathways [36].

In normal ovarian tissue, collagen fibers are thin and long while in tumor stroma they are thicker and shorter fibrils perpendicularly aligned rather than parallel to the tumor boundary [37]. Elevated collagen deposition and remodeling promote tumor progression and drug resistance [10]. Collagen type I enhances the migration of multiple cancer cell lines due to increased directionality [38]. At early stages, collagen types I and III are widely distributed; however, the levels decrease as the disease progresses [39]. During OvCa progression, high levels of collagen type XI have an important role in cell invasiveness, cell proliferation and tumor formation [40]. Collagen type IV and laminin are often absent in benign ovarian surfaces and primary ovarian tumors. At later stages, the restoration of collagen type IV and laminin presence promotes the spread of OvCa cells to metastatic sites in the peritoneum [41,42].

Hyaluronan is a major component of the ECM that has been associated with metastatic progression and poor ovarian cancer outcomes [19,43]. High levels of hyaluronan were detected in metastatic lesions and primary tumors [44] and correlated with increased versican levels [10]. In ovarian cancer, the interaction between hyaluronan and its cell surface receptor, CD44, facilitates cell adhesion, migration, tumor growth and peritoneal dissemination [43,44,45]. Increased expression of fibronectin was found in metastatic tumors and ascitic fluid. Fibronectin is an indicator of poor prognosis that contributes to the formation, adhesion and disaggregation of OvCa spheroids and promotes tumor migration, invasion and metastasis [10,46]. The stroma also contains tenascin-C that contributes to tumorigenesis and metastasis [47]. Tenascin-X is considered as potential biomarker of OvCa as it is highly overexpressed in tumorous tissues [48]. In addition, the downregulation of decorin and lumican are involved in cancer progression and aggressiveness, respectively [10]. As ECM-associated components, MMPs, mediate ECM remodeling and tumor development. Particularly, MMP-9 and MMP-2 facilitate cancer cell invasion via the degradation of collagen IV at the basement membrane [19]. Overexpression of LOX enhances the crosslinking of the ECM proteins resulting in increased matrix stiffness [10].

As outlined above, the bidirectional interactions between cancer cells and the TME are crucial in disease progression. For this reason, it is critical to understand the functions of cancer cells and how the TME regulates the surrounding stroma to promote disease progression. Having briefly outlined the components of the ovarian TME, the next part of our review will summarize selected 3D cancer models engineered to mimic the ovarian TME. These include animal (e.g., rodents, laying hen) and 3D in vitro (e.g., spheroids, organoids, microfluidics, hydrogels) approaches.

## 3. 3D Ovarian Cancer Models

One of the most prevalent challenges in cancer research is the modeling of disease progression and the specific microenvironment that surrounds the tumor. While 2D cell culture has long been used as a platform for cancer research, studies demonstrated that relevant features of the human TME cannot be recapitulated in these 2D systems. Traditional 2D cell culture has many drawbacks, including the absence of the 3D architecture and matrix components, limited cell–cell and cell–matrix interactions, changes in drug responses, limitations to study metastatic processes and gene expression changes with repeated passages [49]. Cells cultured on 2D plastic dishes maintain a flat morphology and organize in a monolayer due to the substrate stiffness. Whereas cells grown in 3D culture have a round morphology and organize in clusters or aggregates [50].

**Figure 2:**
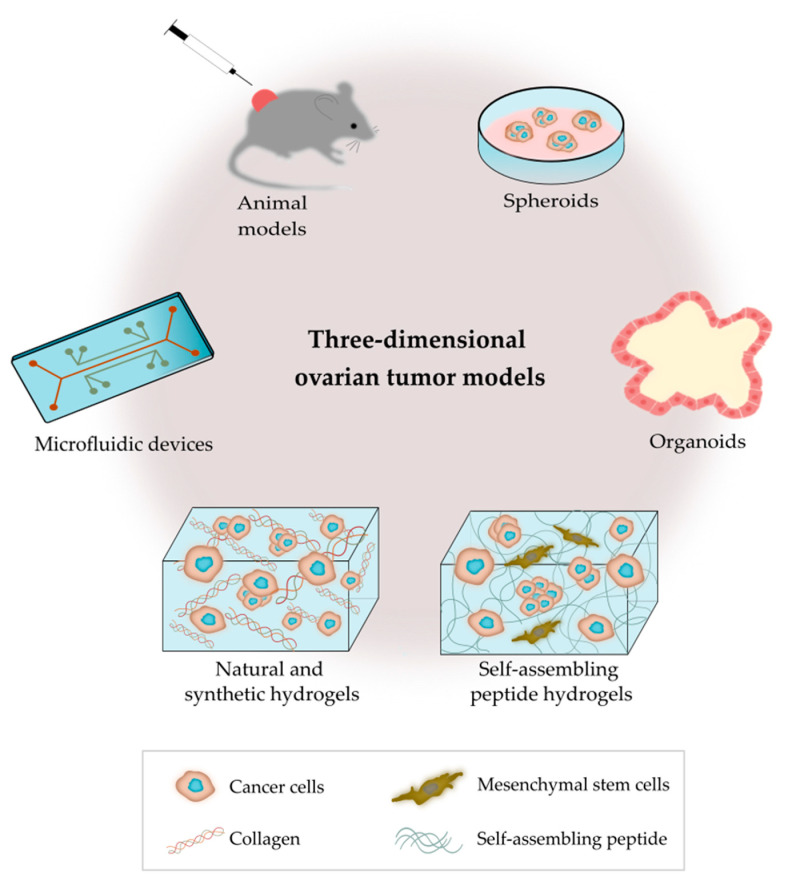
Outline of the main 3D platforms used to model the tumor microenvironment of ovarian cancer.

As outlined in Figure 2, there are several bioengineering approaches to model the ovarian TME. 3D models replicate the characteristics of tumors seen in patients, as they contain multiple cell types and ECM components that interact simultaneously in a complex manner [51]. Bioengineered 3D models also provide the opportunity to mimic the heterogeneity of ovarian tumors, and important biomechanical properties, such as matrix density and stiffness [52]. 3D cancer models are critical tools for the study of tumor biology, metastatic pathways and drug screening [53].

### 3.1. Animal Models

#### 3.1.1. Rodent Models

Rodents are the most widely used animals for in vivo modeling for xenograft (human to murine) approaches, as syngeneic (murine to murine) and genetically-modified models [54,55,56]. Patient-derived xenografts (PDXs) are commonly used to generate tumor models given their capability to resemble a tumor’s phenotype and genotype, the formation of ascites and vasculature, metastatic potential and clinical response to chemotherapy [57,58,59]. PDX models are a research tool for drug development, improving our understanding of tumor biology and for studying biomarkers of therapy response and resistance [60,61]. The advantage of PDX models is the establishment of tumor banks that have previous treatment results and preclinical trial data to optimize therapy success [62,63]. The main limitation associated with the use of PDXs is the displacement of the tumor graft inside the murine host. Zhang et al. implemented the use of a hemostatic gauze to wrap the ovary and provide a physical barrier [57]. Other limitations are the low success rate of PDXs, high cost and long processing times [64]. An additional challenge is the replacement of the human stroma with murine stroma [65], as well as the lack of human immune elements, which hampers the screening of immunotherapies or interactions between tumor cells with the immune system [58,66].

Syngeneic mouse models of OvCa are established from murine ovarian surface epithelial cells (e.g., ID8 cells) that are injected back into the mice to evaluate tumor growth and metastasis [67]. Interestingly, syngeneic models show extensive metastasis in the peritoneal cavity accompanied by the formation of ascites, indicative of disease aggressiveness and tumorigenicity [68,69]. The intact immune system in syngeneic mouse models allows studies of immune response to new therapeutics or tumor progression. However, these models are based on animal-derived cells and not human cells or tissues [55].

Genetically-engineered mouse models (GEMMs) comprise mice altered by genetic engineering techniques [70]. In OvCa, our limited understanding of the disease and its heterogeneity make it difficult to establish GEMMs [67]. Thus, GEMMs are often used to study the initial stages of OvCa or metastasis and to analyze the functions and interactions of oncogenes and tumor suppressors during these processes [70,71]. GEMMs display the genetic heterogeneity, the molecular and histopathological features of the primary tumor and offer flexibility through genetic manipulation [72,73,74]. Zhai et al. established a GEMM to show that different combinations of genetic alterations in the fallopian tube epithelium lead to different tumor phenotypes [75]. Although GEMMs are developed in a natural microenvironment, they still include mice with a different metabolism compared to humans. GEMMs are costly and time-consuming [58], and the promoters employed in these models are scarce for the study of OvCa [70].

#### 3.1.2. Laying Hen Model

The laying hen model has been used as an alternative to rodent models for the study of OvCa progression. The hen is an experimental model that develops spontaneous OvCa without the need for genetic or chemical manipulation [76]. Hen tumors are similar to human tumors in their pathological and genetic characteristics, malignancy, developmental pattern and dependence on age and number of ovulations [54,77]. Using the laying hen model, it has been demonstrated that the oviduct may be the tissue of origin for OvCa [78,79]. The main advantage of this model is the high incidence of the disease [80]. There are still many technological limitations that hinder its widespread application, such as the lack of species-specific antibodies or knockout technologies for the inactivation of genes and pathways in chickens, making rodent models more appealing for cancer research [76,80]. Furthermore, the lack of mechanical characterization of the tumors limits the relevance of the present strategy.

Overall, animal models are not ideal for modeling all stages of OvCa development and progression. The application of animal models in preclinical research has diminished as mice do not develop OvCa spontaneously. Animal models lack stromal components that are fully representative of human tumors. For these reasons, the application of 3D in vitro models in cancer research has emerged as a novel approach to create more biologically relevant models that recapitulate the complexity of the TME, avoiding the costly, labor-intensive and ethical concerns of using animals for research (Table 1).

### 3.2. 3D In Vitro Models

#### 3.2.1. Spheroids

Spheroids are cell aggregates that maintain uniform geometry and chemical gradients (e.g., oxygen, nutrients and metabolites) at diameters ranging from 200–500 µm, forming a necrotic core at sizes > 500 µm [128]. Different techniques have been used to generate spheroids, such as suspension culture, non-adherent culture, hanging drop culture, microfluidics and matrix encapsulation [129,130]. Cells grown as spheroids have a similar nutrient transport, growth kinetics and cell–cell interactions to that reported in solid tumors [131]. Particularly for OvCa, multicellular tumor spheroids (MCTs) are used to mimic the tumor cell aggregates found in ascites [132,133]. MCTs are derived from the aggregation of single tumor cells (homotypic spheroids) or 3D co-cultures (heterotypic spheroids) with different ratios of cancer and stromal cells (e.g., CAFS, CA-MSC or macrophages) [81,82,134,135].

**Figure 3:**
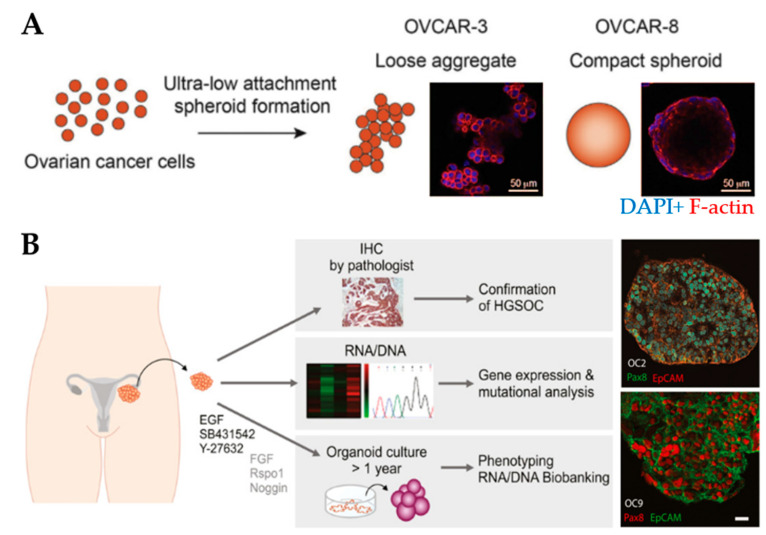
Spheroid and organoid cultures to model the ovarian tumor microenvironment. (**A**) An example of the formation of loose and compact aggregates using OVCAR-3 and OVCAR-8 cells, respectively. Samples were stained at the end of 6 days using F-actin (red) and nuclear marker DAPI (blue) (Reprinted from [85], Copyright © (2020), with permission from Elsevier). (**B**) An example of the establishment of organoids derived from patient-derived xenografts from tumor deposits in the peritoneum. Organoids express the high-grade serous ovarian cancer marker Pax8 and the epithelial cell adhesion molecule EpCAM. Scale bar: 20µm (Reprinted from [93], Copyright © (2020), with permission from John Wiley and Sons).

Spheroids are the preferred choice for studying drug efficacy as they show greater resistance to therapies (e.g., paclitaxel and cisplatin) compared to 2D cell cultures [136,137], for example, through the increased expression of stemness-associated genes [83]. MCTs have been used to understand the relationship between the degree of spheroid aggregation, tumor progression and drug resistance. Spheroids derived from OV-90 and OVCAR-3 clusters showed greater resistance to anoikis compared to spheroids derived from single-cell counterparts [84]. OVCAR-8 cells formed compact spheroids with high migratory capacity compared to the loose aggregates obtained from OVCAR-3 cells (Figure 3A). Moreover, spheroids displayed size-independent resistance against anti-cancer drugs [85]. Nectin-4 peptides inhibited the formation of OVCAR-5 and CAOV3 spheroids, suggesting that their addition to chemotherapeutic agents may increase the efficacy of maintaining cells as single cells or small aggregates [86]. Another study reported that the addition of laminin-1-derived synthetic peptide AG73 promoted the formation and growth of spheroids [138]. The addition of scrambled AG73T peptide to laminin-1-stimulated spheroids caused their disaggregation and induced apoptosis by cisplatin. Thus, scrambled AG73T peptide in combination with cisplatin may represent an improved therapeutic strategy.

MCTs were found to express different genes compared to single cells, which influence the responses to drugs [139]. Spheroids derived from OvCa cell lines following treatment with anti-cancer drugs (e.g., cisplatin, paclitaxel and topotecan) upregulated genes that are associated with cell proliferation, cellular assembly and organization, cell death and cell cycle control [87]. MCTs had a unique expression pattern, and their cell cycle pathway was altered compared to 2D cell monolayers, with CDC25A, a promoter of resistance to anti-cancer drugs, being upregulated [88]. CD73 was found to be related to sphere formation, stemness and EMT-associated genes [89]. Spheroids enriched with CSCs acquired a quiescent phenotype with increased stem cell markers (e.g., ALDH1, CD133, CD24 and SOX2) and suppressed cell adhesion and cell cycle markers [90,91,92].

Overall, the application of MCTs has been increasing in the last decades as they more closely resemble solid tumors compared to 2D models. Spheroids are suitable for anti-cancer drug discovery and screening. Their main advantages are their low cost, ease of use, reproducibility and suitability for high-throughput assays [135]. However, when using MCTs, future improvements include an increased resemblance to solid tumors and their microenvironment, for example, the tumor vasculature, components of the immune system, mechanical signals and fluid dynamics [9,135,140]. It is crucial to overcome the difficulty to acquire high-resolution images of drug penetration and cell invasion through spheroids because of the loss of image quality, poor light scattering and lack of compartmentalization between cancer and stromal cells [9,130,134,140].

Standardized protocols need to be established to assist researchers in generating MCTs with uniform sizes and for high-throughput assays [129,135]. A major limitation is that not all cell lines are capable of forming spheroids or aggregates, while those that generate spheroids can present different morphologies depending on the technique (Table 1), thus, affecting their migratory capacity and chemoresistance [85,133,141,142]. The addition of a matrix using scaffolds (e.g., hydrogels) will allow spheroids to develop their own ECM and create more relevant 3D models similar to the tumors seen in patients.

#### 3.2.2. Organoids

Organoids are referred to as 3D structures derived from either adult or pluripotent stem cells [143,144]. Tumor-derived organoids are grown from primary and metastatic tumors, pleural effusion drainage, ascites, normal fallopian tube and ovarian surface epithelium [94,145]. Organoids have been employed as an intermediate model between cancer cell lines and xenografts [146].

The organoid platform retains the physiological (e.g., architecture and intercellular communication) and genetic features similar to the original tumor (Figure 3B) [146,147]. The platform recapitulates the intra-tumor heterogeneity found in solid tumors, including OvCa, a feature that causes variations in treatment responses between patients [93,94,95,148]. Organoids can be genetically modified, expanded for long-term culture and cryopreserved [56]. Several organoid biobanks have been established to allow the testing of novel drugs and predict patient response prior to treatment [93,146,149,150]. The development of these resources is costly but less time-consuming compared to PDX models [150].

Recent studies indicated the versatile applications of organoids from diverse sources. Nanki et al. established organoids from clear cell OvCa resistant to platinum-based drugs, paclitaxel and Olaparib, and organoids harboring BRCA1 and sensitive to cisplatin and Olaparib [96]. Organoids grown from MCTs retained the features of malignant ascites [97].

Tumor-derived organoids have been used in cancer research for drug screening as an alternative to explore genetic alterations that cause therapy resistance [98,99]. Genomic analysis revealed that drug sensitivity was related to DNA repair deficiency in the organoids [100].

Organoids are a great tool for the study of tumor biology, but there are several limitations in their use (Table 1). They lack components of the immune system, stromal cells and vasculature, which is restrictive for therapeutic screening [56]. The use of microfluidic devices can overcome this challenge, as they facilitate the co-culture of tumor-derived organoids with other stromal or immune cells to recreate a more complex TME [151]. Organoids are more costly compared to 2D cell cultures due to supplemental factors required for their culture, and the addition of growth factors and other molecules may affect the natural morphogen gradients found in tissues [152]. The intra-tumor heterogeneity may be lost during passages causing subsequent clones to grow differently. Mutations can also be acquired during long-term expansion [143]. Moreover, culture protocols and drug screening strategies vary among laboratories and research studies, possibly affecting the outcome of organoid-based assays [143,152]. Overall, organoids are a versatile tool for cancer research, drug discovery and screening.

#### 3.2.3. Microfluidic Devices

One of the major bottlenecks of 3D in vitro models is the limited recreation of the dynamics found in the TME. For that matter, microfluidic devices are a revolutionary technology that addresses this challenge. These devices consist of microwells connected by channels with different geometries that can include perfusion (e.g., intermittent, cyclic or continuous), shear stress, nutrient delivery and waste removal [153,154,155,156]. Microfluidic devices can be used to perform drug screening with the advantage that the drugs can be combined and the respective concentrations can be adjusted [157]. Researchers have developed a variety of microfluidic devices to model diverse organs or tissues, such as the breast [158,159], liver [160], intestine [161,162], heart [163], lung [164,165,166] and skin [167].

Cells grown in microfluidic devices, also known as organ-on-a-chip devices, can be implemented to create tumor-on-a-chip models to investigate tumor development, metastasis and drug responses [154,168]. Unlike static cultures, microfluidic devices recreate important aspects of the TME, such as vascularization, invasion and migration of cancer cells, while maintaining cell viability over a long time (e.g., days to several weeks) [169,170]. Moreover, microfluidic devices allow the addition of different cell types within separate chambers to create a more complex TME. This 3D co-culture system provides a new way of understanding cell–cell interactions through culture medium diffusion between chambers [171]. Li et al. developed a microfluidic device containing OvCa spheroids in contact with human mesothelial cells to resemble the metastatic process within the peritoneal cavity [102]. However, this device did not ensure the control of individual nutrient replenishment and shear stress.

**Figure 4:**
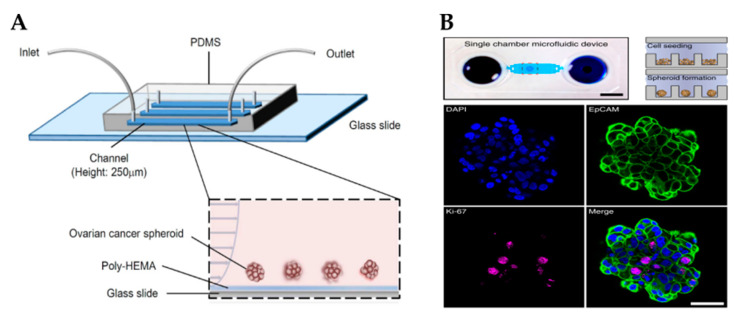
Microfluidic platforms to grow ovarian cancer spheroids. (**A**) Schematic showing a poly-HEMA-coated microfluidic channel for the formation of ovarian cancer spheroids under perfusion (Reproduced with permission from [83], Copyright © (2016), Springer Nature). (**B**) An example of a microfluidic chamber containing an array of microwells for spheroid formation (**top**). Scale bar: 5 mm. Spheroids were stained for the epithelial marker EpCAM, proliferation marker Ki-67 and nuclear marker DAPI (**bottom**). Scale bar: 50 µm (Reproduced with permission from [104], Copyright © (2020), Springer Nature). Poly-HEMA, poly 2-hydroxyethylmethacrylate; PDMS, polydimethylsiloxane.

Cells within the OvCa niche experience a range of mechanical stimuli, such as shear stress, compressive stress, matrix stiffness, tensile stress and stress relaxation, which influence cancer progression, response to drugs and metastasis [109]. Several strategies have been developed to add biomechanical cues into microfluidic devices, such as the application of shear stresses or compression forces, to better study cancer progression and chemoresistance. For example, Onal et al. created a microfluidic device integrated with actuators to apply compression on SKOV-3 cells [172]. It has been reported that cells under compression stimulation displayed invasive morphology, increased proliferation and chemoresistance [173]. Rizvi et al. created a microfluidic model with OVCAR-5 cells using continuous laminar flow [103]. Different from cells grown in static conditions, in fluidic conditions, OvCa cells formed micronodules with an EMT-like phenotype (e.g., downregulation of E-cadherin and upregulation of vimentin), increased motility and metastatic potential. Another study reported that SKOV-3-derived spheroids grown under shear stress acquired an EMT-like phenotype, expressed CSC markers and were resistant to cisplatin and paclitaxel through the activation of ABCG2 and P-glycoprotein (Figure 4A) [83].

As cancer progresses, the remodeling of the ECM causes tissue stiffening that affects tumor development, metastasis and chemoresistance [37]. In the design of the microfluidic devices, the stiffness of the surface must be considered, as it plays an important role in the behavior of cancer cells [174]. For instance, OvCa cells on soft substrates presented resistance to chemotherapeutic drugs through a mechanosensitive Rho/ROCK pathway [37,175]. The influence of matrix stiffness on OvCa cells using microfluidic devices has not been studied in detail and this remains unclear. However, studies have reported the creation of microfluidic devices with variable matrix stiffness to study their effect on tumor cells. For example, Anguiano et al. created a platform filled with collagen/Matrigel hydrogels of different concentrations to analyze cancer cell migration [176]. Another device with defined wall stiffness and geometry was created to allow independent variation of ECM stiffness and channel width [177]. These models suggest that the addition of variability in matrix stiffness could greatly enhance the ability of future devices to study OvCa progression and chemoresistance depending on the conditions of its microenvironment.

Due to the high resistance to chemotherapy exhibited by OvCa, there is an imminent need for more effective therapeutic strategies for its treatment. For instance, combinatorial targeted therapy is a potential tool to use drugs at lower doses, reduce side effects and overcome drug resistance presented by cancers with a heterogeneous structure [178]. Flont et al. compared the effect of sequential combination therapy (chemotherapy with doxorubicin and photodynamic therapy) against OvCa cells (A2780) and human ovarian fibroblasts under static and dynamic conditions [105]. It was proven that the use of dynamic conditions and sequential drug delivery improved the effectiveness of the treatment. Another study reported a microfluidic print-to-screen platform that integrates combinatorial therapy for high-throughput parallel drug screening [106]. SKOV-3 cells were cultured within agarose gels and placed inside the microfluidic platform where they were subjected to distinct drug combinations. Overall, 15 drug combinations were identified to have potent cytotoxic properties. Dadgar et al. demonstrated the potential of a serial and parallel perfused multi-chamber microfluidic device to test various drug concentrations on spheroids generated by low cellular input (Figure 4B) [104].

Microfluidic platforms ensure the formation of tumor spheroids with precise control under continuous perfusion. However, the collection of spheroids from these devices can be difficult, compared with other techniques [154,179]. Marimuthu et al. set up a microfluidic device for automatic multi-sized spheroid formation with independent cell seeding densities between pinholes [107]. This model was feasible not only for spheroid formation but also for treatment, immunofluorescent staining and confocal imaging on-a-chip.

Although tumor-on-a-chip devices have demonstrated impressive results for the screening of anti-cancer drugs, compared to animal models, they carry several limitations (Table 1) [180]. These devices are usually more complex to design and use than other 3D systems. Another limitation is the material used to construct the microfluidic devices (polydimethylsiloxane, PDMS). Although PDMS is the most common material due to its excellent optical transparency and flexibility, it can lead to sample absorption, channel deformation under pressure driven by flow, leaching and channel swelling when exposed to solvents such as acetone [181]. The swelling of microfluidic channels results in inaccurate measurements of flow rates [182]. Hence, it is essential to develop novel materials that can be flexible, optically clear and minimally absorptive of drugs or nutrients [183].

Photolithography is widely used for chip fabrication, but it is costly, time-consuming and not readily accessible in most labs. In addition, the majority of the studies on tumor-on-a-chip uses relatively simple biomaterials, such as ECM proteins, to support cell growth. There is a need for more sophisticated biomaterials to facilitate the full potential of microfluidic devices. For instance, biomimetic materials are needed that allow cell growth and tuned mechanical properties according to the organs they are mimicking.

As presented, microfluidic devices possess defined characteristics that make them a relevant tool in cancer research. In the future, a ‘human-on-a-chip’ that integrates multiple chips with different organ functions into one chip is an appealing concept for cancer research, drug testing and the development of personalized treatments [168]. This would provide a pathway to recapitulate the entire TME including the metastatic process to various organs combined with a vascular network.

#### 3.2.4. Hydrogels Based on Polymer/Protein Networks

In an attempt to improve 3D in vitro models, cancer cells have been embedded within hydrogels that mimic the 3D architecture and properties of the ECM [49,135]. Hydrogels consist of crosslinked networks of hydrophilic polymer chains that contain a high amount of water and maintain a distinct 3D structure [8]. They are designed with a broad range of compositions, biological functionalities and mechanical properties [184]. Major advantages of using hydrogels for 3D cell cultures are their ability to incorporate key components of the ECM, including proteins and growth factors, nutrient gradients, and promote cell–cell and cell–matrix interactions. [185]. Therefore, hydrogels have very promising applications for developing 3D in vitro models for cancer research and drug screening.

##### Natural Hydrogels

Hydrogels can be categorized according to their polymeric origin, from natural or synthetic biomaterials. Natural biomaterials (e.g., collagen, Matrigel, fibrin, alginate and hyaluronic acid) have been widely used to engineer 3D in vitro models because of their biocompatibility, increased potential for supporting cell viability and capacity to recapitulate the ECM [186].

Matrigel is recognized as the golden standard for ECM scaffolds because it enables cell–matrix interactions, stimulates spheroid formation and promotes the cell growth of stromal and cancer cells [187]. However, its murine origin, high batch-to-batch variability and undefined composition (Table 1) limit its use in experimental studies and drug discovery [143,188]. As an alternative to Matrigel, injectable alginate-based hydrogels supplemented with laminin or hyaluronic acid have been reported as a method for tumor inoculation [189].

**Figure 5:**
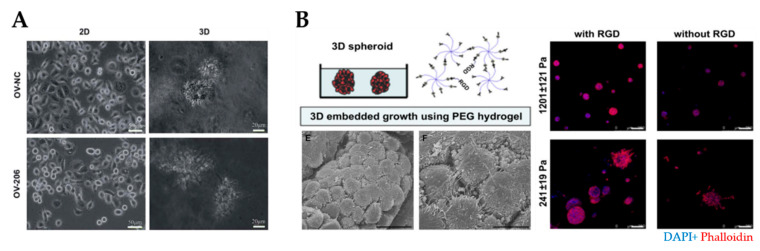
Natural and synthetic hydrogels for modeling of the ovarian tumor microenvironment. (**A**) An example of tumor spheroids formed in 3D collagen scaffolds but not in 2D monolayer culture (reproduced from [38] with permission from the Royal Society of Chemistry). (**B**) An example of PEG/RGD-functionalized hydrogels that supported the formation of spheroids derived from ovarian cancer cells. SEM image demonstrated spheroids formation within the hydrogels (**bottom left**) Cells within spheroids were connected by the development of lamellipodia. Scale bar: 20 µm. Shape and cell spheroid formation varied with the hydrogel stiffness and RGD functionalization: compact and smaller spheroids were obtained in stiffer (G’= 1201 ± 121 Pa) hydrogels, irregular and scattered spheroids grew in softer (G’= 241 ± 19 Pa) hydrogels (**right**). Scale bar: 100 µm. (Reprinted from [113], Copyright © (2010) with permission from Elsevier). PEG, polyethylene glycol; RGD, arginine–glycine–aspartate.

Since collagen is a predominant component of ovarian ECM, and its presence is associated with chemoresistance, collagen-derived scaffolds have been widely used to model the TME [32]. These gels exhibited high chemoresistance, the viability of spheroids and the growth of diverse OvCa cell lines (e.g., OV-NC, OV-206, SKOV-3 and OVCAR-3) (Figure 5A) [38,109,110,111]. Collagen-derived scaffolds not only enhance the invasive and mobile capabilities of cancer cells but also stimulate an EMT-like phenotype [38,111].

Despite the advantages of hydrogels based on natural materials, several drawbacks include their complex purification, limited chemical modification and flexibility to manipulate their mechanical properties [51,190]. The complex molecular composition, batch-to-batch variation and uncontrolled degradation of these materials make it difficult to decipher the interaction of the ECM with cancer cells [186]. To overcome these drawbacks, synthetic hydrogels have been developed.

##### Synthetic Hydrogels

Synthetic polymers, such as polyethylene glycol (PEG), gelatin methacryloyl (GelMA) and poly(lactide-co-glycolide) (PLG), offer several advantages over natural materials including the tunability of their mechanical and biochemical properties. As these types of materials do not contain biological moieties, they must be functionalized with cell adhesion ligands and crosslinkers to encourage cancer cell growth, spreading and migration [191]. Synthetic hydrogels can also be designed to incorporate functional domains of ECM proteins or proteolytic degradation sites [192].

One of the most common synthetic polymers in cancer research is PEG, which can be used to design hydrogels modified with heparin, fibrinogen, IKVAV, RGD and MMP-degradable motifs (Figure 5B) [113,193,194,195,196]. Researchers have taken advantage of the tunability of synthetic hydrogels to investigate the effect of matrix stiffness on cancer progression [114]. Irregular and scattered spheroids were obtained in soft (241 ± 19 Pa) hydrogels, while compact and dense spheroids were formed in stiff (1201 ± 121 Pa) hydrogels [115]. A high elastic modulus (G’ = 12.0 kPa) of PEG-crosslinked poly (methyl vinyl ether-co-maleic acid) (PMVE-co-MA)- based hydrogels contributed to cell adhesion, migration and invasion [116]. Interestingly, SKOV-3 MCTS disaggregated in stiff polyacrylamide hydrogels through mechano-transduction pathways [35]. In the case of soft GelMA-based hydrogels (G’ = 0.5 kPa), loose cell aggregates formed, while stiff hydrogels (G’ ≥ 7 kPa) inhibited cell proliferation and led to smaller cell aggregates with decreased metabolic activity [117].

Synthetic hydrogels have been employed as an alternative option for MCTS as these scaffolds not only support 3D cell organization but also mediate cell–cell and cell–matrix interactions. Lee et al. established microwells using PEG-based hydrogels to generate homogeneous and uniform-sized MCTS using OvCa cells [118]. Loessner et al. reported a similar microwell array to assess the formation of OV-MZ-6 MCTS and their survival with paclitaxel treatment [119]. The application of synthetic hydrogels to produce tumor spheroids with reproducible and homogeneous size represents a great potential approach for drug screening.

#### 3.2.5. Hydrogels Based on Self-Assembled Peptide Networks

Molecular self-assembly is an appealing approach for the bottom-up fabrication of nanostructured biomaterials with unique mechanical and chemical properties. Self-assembly is defined as the spontaneous aggregation of individual molecules into higher-ordered structures driven by non-covalent interactions, such as hydrophobic effects, hydrogen bonding, van der Walls, aromatic stacking and electrostatic forces. Nature uses self-assembly to create complex biological structures ranging from cell membranes, proteins and DNA molecules to viruses [197]. The use of peptides as building blocks for functional nanomaterials offers the possibility to synthesize self-assembling models with a higher level of control and specific intermolecular interactions, which can be modified by varying the peptide sequence. These adaptable systems are very attractive for 3D cell cultures because of their composition, structural and mechanical similarity to the native ECM [198]. Other advantages of self-assembling peptide materials are their versatile synthesis, biocompatibility, fast gelation and bioactivity to promote cell–cell and cell–matrix communication [199]. The mechanical properties of self-assembling models can be precisely tuned by modifying their sequence or adjusting their concentration or crosslinking density via different (ionic) mechanisms [192,200]. Peptide sequences can be designed to contain functional epitopes to enhance bioactivity and customize the interaction of cells with the matrix [51]. In addition, peptide sequences can be used as drug delivery vehicles [201]. Hurley et al. reported the development of a self-assembled microscale carrier conjugated with the peptide WSGPGVWGASVK entrapping the chemotherapeutic drug topotecan. Controlled drug release affected cell proliferation, mitigated cell migration and induced the loss of lamellipodia in SKOV-3 cells. All these features enhance the functionality of peptide hydrogels to support cell functions, such as survival, proliferation, migration, adhesion, invasion and differentiation. Thus, self-assembling hydrogels are used in diverse areas including regenerative medicine [202], tissue engineering [203], cancer research [8] and drug and gene delivery [204].

The building blocks of self-assembling peptides are classified by their different constituent amino acids and bound chains or motifs including dipeptides, surfactant-like peptides, peptide amphiphiles, bolaamphiphilic peptides, ionic complementary self-assembling peptides and cyclic peptides [202]. In the context of modeling the ovarian TME, ionic self-complementary peptides and peptide amphiphiles have received considerable attention and will, therefore, be discussed in more detail in the next sections. Readers are referred to several reviews [197,202,203,205] for more details on self-assembling peptides.

##### Ionic Complementary Self-Assembling Peptides

Zhang et al. [206] first designed the ionic complementary self-assembling peptides (AEAEAKAKAEAEAKAK, EAK 16-II) in 1993. These peptides are based on hydrophobic residues on one side and hydrophilic residues on the opposite side containing an alternating arrangement of negative and positive charges. They self-assemble into stable β-sheet structures in solution and then form nanofibrous porous hydrogels resembling the architecture of the native ECM.

**Figure 6:**
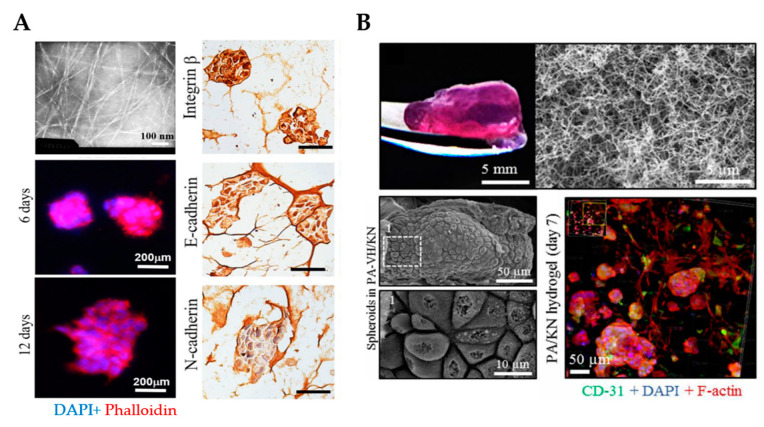
3D culture of ovarian cancer cells in self-assembling peptide scaffolds. (**A**) An example of self-assembling RADA16 hydrogels with a fibrous network that supports spheroid formation and growth for up to 12 days. Transmission electron microscopy was employed to analyze the nanofiber structure of RADA16 in solution (**top left**). Spheroids formed in hydrogel matrices were imaged on days 6 and 12 using phalloidin (red)/DAPI (blue) staining (**bottom left**). Immunohistochemistry images show the cell distribution and molecular expression of integrin β1, E-cadherin and N-cadherin in cells cultured in RADA16 hydrogels for 7 days. Scale bar: 200 µm. (Reproduced with permission from [125], Copyright © (2020), Springer Nature). (**B**) An example of PA/KN hydrogels with an internal heterogenous nanofibrous structure that permitted spheroid formation and growth for 14 days (**top**) SEM images demonstrate the growth of tumor spheroids within PA-VH/KN hydrogels on day 14 (**bottom left**). Immunofluorescence staining of CD31, F-actin network and nuclei in PA/KN hydrogels on day 7 (**bottom right**). From [127]. Reprinted with permission from © (2020), AAAS. PA-VH, C_16_VVVAAAVPGIGH_2_K; KN, keratin.

RADA16 (Ac-RADARADARADARADA-CONH_2_) is a representative of the most widely used ionic complementary self-assembling peptides, which is commercially available as PuraMatrix™. The basic molecular building block of PuraMatrix™ is a tetrapeptide containing arginine–alanine–aspartate–alanine (RADA) residues that are similar to the RGD cell adhesion sequence. PuraMatrix™ self-assembles to form highly organized scaffolds that facilitate the proliferation and differentiation of diverse OvCa cells (e.g., OVCAR-5, A2780, A2780/DDP, and SKOV-3) [123,124]. RADA16 scaffolds promoted tumor spheroid formation and cell viability for up to 12 days (Figure 6A) [125,126]. When compared to 2D cell cultures, cells grown in RADA16 hydrogels showed higher resistance to multiple therapeutic drugs (e.g., paclitaxel, cisplatin, 5FU, and curcumin) [125,126]. These peptide hydrogels form nanofiber networks with pore sizes ranging from approximately 5–200 nm, which closely mimics the porosity of the native ECM [124]. In addition, RADA16-I scaffolds recapitulate similar functionalities to collagen gels as cancer cells adhere and invade into the matrix.

A similar molecular design was used to produce a new family of self-assembling polypeptide hydrogels with tunable mechanical properties, commercially available as PeptiGels^®^ from Manchester BIOGEL. These hydrogels are suitable scaffolds to grow pancreatic cancer Suit2 cells with defined conditions (e.g., pH, stiffness and temperature) [207]. PeptiGels^®^ have also been used to grow and expand breast cancer MCF-7 and MDA-MB-231 cells, allowing the recreation of hypoxic and invasive conditions found in solid tumors [208]. While MCF-7 cells formed large and compact spheroids resembling acini, MDA-MB-231 cells remained dispersed. These studies highlight the increasing use of self-assembling peptides in cancer research to model the TME of different cancers.

##### Peptide Amphiphiles

**Figure 7:**
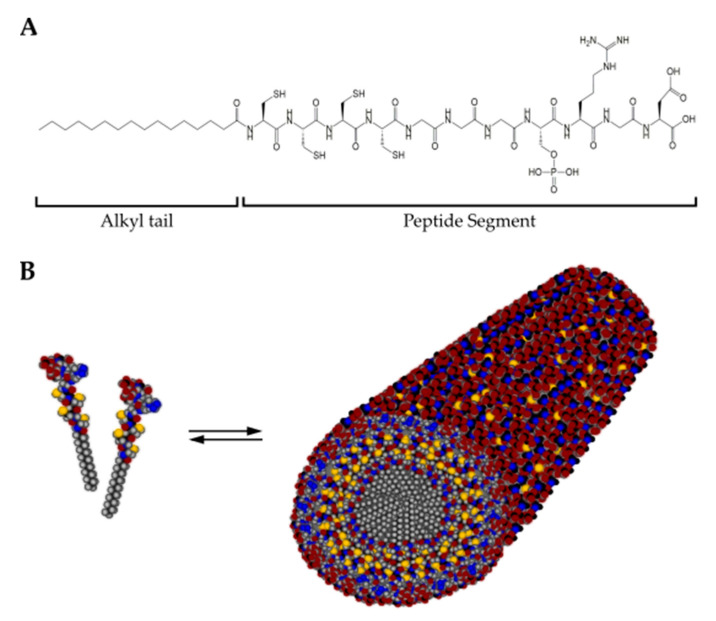
(**A**) Chemical structure of a representative peptide amphiphile (C_15_H_31_CONH-CCCCGGGS(P)RGD-OH) encompassing a hydrophobic alkyl tail and the peptide segment. (**B**) In solution, peptide amphiphile molecules self-assemble into cylindrical micelles with a hydrophobic core surrounded by the peptide segment. Color scheme: Carbon, black; Hydrogen, gray; Oxygen, red; Nitrogen, blue; Phosphorous, cyan; Sulfur, yellow.

Peptide amphiphiles (Pas) are defined as peptide-alkyl-chain surfactants that contain a peptide sequence covalently bound to a hydrophobic segment (Figure 7). Under physiological conditions, they self-assemble into high aspect ratio nanofibers that form a network similar to the native ECM [197,209]. PAs can present a variety of bioactive epitopes at the surface of the self-assembled structure to direct cell processes. For example, RGDS motives can be attached to stimulate cell adhesion [210], GHK to enhance migration [211] and IKVAV for neural differentiation [212]. In addition, ECM components, such as keratin, fibronectin and hyaluronic acid, are incorporated into the self-assembling systems to increase the resemblance with in vivo tissues [127,213]. Applications of PAs include, for example, angiogenesis [214], regeneration of cartilage [215,216], enamel [217], skin [213], bone [210,218], and cornea [219]. For a detailed description of PAs, readers are referred to [203,220,221].

Despite not yet fully exploited, PAs are also of particular interest to create 3D in vitro models for cancer research. Our team recently reported an approach comprised of PAs co-assembled with ECM macromolecules for the 3D co-culture of OVCAR4, human mesenchymal stem cells (hMSCs) and human umbilical vein endothelial cells (HUVECs) to study tumor growth and progression (Figure 6B) [127]. Major advantages offered by this system are the formation of a nanofibrous network that resembles the ECM and the incorporation of relevant proteins of the ovarian TME, such as fibronectin or keratin (KN). Given that the hydrogel stiffness influences cell behavior, the PA/KN hydrogels were engineered to exhibit a stiffness within the range (G’ > 0.5 kPa and G’ < 7 kPa) reported to support tumor spheroid formation [117]. Cell-containing PA/KN hydrogels remained stable for up to 28 days. The cross-sectional size of the tumor spheroids was compared to previous studies [113] and exhibited cell anchorage to the nanofibrous network. PA/KN hydrogels supported high cell viability of all three cell populations including hMSCs and HUVECs. In 3D co-cultures, cell spreading occurred due to the interaction between the OvCa cells with hMSCs and HUVECs. Importantly, spheroid size and growth were enhanced in 3D co-cultures compared to the 3D mono-culture counterparts. The incorporation of bioactive peptide sequences (e.g., RGDS and GHK) promoted the formation of an extensive F-actin network, which was absent in 3D monocultures. This network was established throughout the self-assembling matrix and intercalated between adjacent spheroids. As a proof-of-concept, PA/KN hydrogels were used to evaluate cell responses to drug treatment. Both paclitaxel and carboplatin treatment prevented tumor spheroid formation and the remaining cells exhibited a low metabolic activity.

Other advantages of this innovative platform are the capacity to improve reproducibility and minimize batch-to-batch variations, well-known limitations of Matrigel used for 3D cultures. PAs form a nanofibrous network, which is not the case for PEG-based and GelMA-based systems [113,117]. Importantly, PA/KN hydrogels have been previously employed for 3D bioprinting, proving an exciting new opportunity to incorporate cells with spatial control for biomedical applications [222]. The limitations of PAs are the high cost of these materials and low scalability (Table 1). Alternatively, hydrogels made of shorter and simpler self-assembling peptides, such as Biogelx™, can potentially be used as cell culture scaffolds due to their lower cost and simpler synthesis. These short (di- or tri-) peptides (e.g., diphenylalanine), modified at the N-terminus with aromatic fluorenylmethoxycarbony (Fmoc) group, self-assemble into nanofibers in aqueous conditions that entangle in the presence of Ca^2+^ ions to form hydrogels [223]. Biogelx™ hydrogels support the viability of a number of cell types, including human/bovine chondrocytes, human dermal fibroblasts, human bone marrow stem cells and human adipose-derived stem cells. Biogelx™ has been investigated for use as 3D in vitro model for anti-cancer drug testing using breast cancer MCF-7 cells [224].

Given the advantages and opportunities that PA systems provide and the need to better recreate the ovarian TME, we consider that self-assembling hydrogels represent an exciting 3D approach that will further advance the development of this technology. Overall, the ability to co-culture multiple cell populations within this system, that also contains bioactive epitopes and ECM proteins, demonstrates the relevance of this 3D approach to recreate the ovarian TME. These features were also exploited by our team for pancreatic cancer research [225]. Pancreatic cancer and stromal cells (CAFs and macrophages) were grown embedded in self-assembling hydrogels that also contained specific ECM molecules to test a combination of anti-cancer drugs (e.g., gemcitabine, nab-paclitaxel and triptolide). Another study reported the engineering of self-assembled PA-PEG composite hydrogels functionalized with RGD and DGEA (Asp-Gly-Glu-Ala) [226]. After tuning the mechanical properties by changing the PEG concentration, human osteosarcoma cells were grown within this hydrogel.

Upon evaluating the state-of-the-art applications of self-assembling biomaterials in cancer research, the versatility of using this platform as a 3D in vitro model is evident and demonstrates an alternative tool to engineer 3D systems for OvCa. In this regard, the potential next step would be to engineer self-assembled systems that incorporate multiple ECM components (e.g., collagen or hyaluronic acid), which are often overexpressed in OvCa or found in patient-derived ascites. It is important to note that the addition of ascites further favors the recreation of the TME as this tumor fluid promotes disease progression. A fibrous network scaffold may be created by integrating the critical elements that provide bioactivity as well as biological, mechanical and chemical properties to study the tumor biology, metastatic pathways and drug resistance.

#### 3.2.6. Mechanical Stimuli in OvCa Models

**Figure 8:**
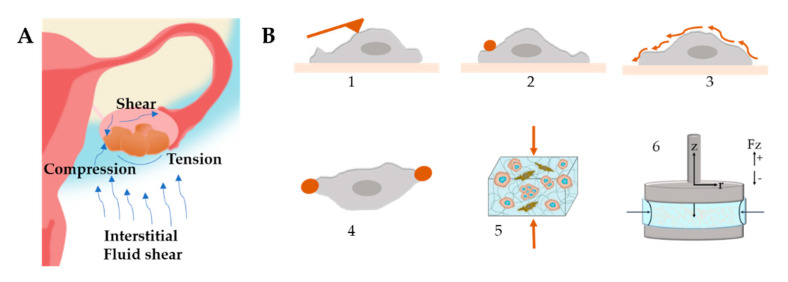
(**A**) Mechanical stimuli found within the ovarian tumor microenvironment that influence tumor progression and metastasis. Ascitic buildup within the peritoneal cavity exposes cancer cells to shear stress. Tumor growth provides radial tension and axial compression to cancer cells. (**B**) Stiffness characterization techniques (1) Atomic Force Microscopy, (2) Magnetic Twisting Cytometry, (3) Shear Flow, (4) Optical Tweezers, (5) Compression Test, (6) Rheology.

In tumor tissues, cancer cells constantly sense physical forces such as mechanical tension, compression and shear stress (Figure 8A). The ECM, in particular, exposes the cells to the increase in matrix stiffness and variable viscoelasticity [227]. These forces can influence cancer progression, metastasis and chemoresistance [36,228]. However, the specific pathways on how they are connected are still not completely understood. Therefore, the study of mechanics in 3D systems and the signaling pathways between cells and the ECM has gained considerable attention. Readers are referred to reviews [229,230] for more details on the effects of ECM on cellular behavior.

Once spheroids have formed, they experience compressive stress against the surrounding stromal compartments as a result of tumor growth [231]. At the same time, the surrounding matrix provides resistance to the expanding cells. Along with the compressive stress, the mechanical forces derived from the matrix can drastically influence the tumor development and the pathways involved. Some models used to study cancer compressive mechanotransduction include compression bioreactors [173] and hydrogels exposed to static compression [232].

The elective techniques to measure cell stiffness (Figure 8B) include optical tweezers, magnetic bead cytometry and atomic force microscopy (AFM) combined with live imaging [233]. Another interesting strategy is the use of beads as stress sensor mechanosensors to capture the internal stress that arises during spheroid formation [232,234]. By using AFM, it was demonstrated that the invasiveness and migratory capacity of cells are correlated with a reduction in cell stiffness [235]. Another study determined that SKOV-3 cells are less resistant to mechanical deformation, which may be a facilitating factor in their metastatic behavior [236]. Conrad et al. determined the mechanical properties of OvCa cells and the effect of chemotherapeutic drug treatment by Brillouin confocal microscopy [237].

In OvCa, tumor cells are under constant shear stress due to the buildup of ascites [227]. Culture under continuous fluid flow using microfluidic devices is an approach to introduce this mechanical stimulus. Shear stress stimulation is known to increase proliferation and chemoresistance, as discussed in Section 3.2.3 Microfluidic devices.

Matrix stiffness not only affects cellular response, differentiation, migration capacity and the cellular response to therapeutics but also the phenotype of cancer cells [227]. The effect of matrix stiffness on cancer cells has been studied using 2D models and 3D models. In most of the studies in 2D, cells are cultured on coated substrates, while the 3D settings use single cells. In the context of multicellular systems, the relationship between microenvironmental stiffness, tumor cell mechanics and invasion has been much less explored despite its importance to cancer progression.

It has been shown that cell stiffness, spheroid size and compaction are altered by matrix stiffness, matrix degradability and variability in compressive stress levels [234,238]. Compression testing and AFM were used to determine that MCTs became stiffer when subject to stiff microenvironments [234]. Spheroids tended to be smaller, more compact and less proliferative when in contact with a stiff environment.

In general, fibrous hydrogels show two main nonlinear mechanical behaviors, namely strain stiffening and negative normal stress difference when shear is applied [239]. These behaviors depend on the specific mechanical properties of the individual fibers or from the network topology. The mechanical properties of the matrix are typically measured with oscillatory rheology (Figure 8B), where stiffening is detected during the amplitude strain or stress sweep as a sudden increase in a modulus beyond a certain stress or strain value.

Cancer cells can recognize the changes in matrix stiffness and respond by generating increased traction forces on their surroundings by actomyosin and cytoskeleton contractibility. Changes in matrix rigidity are sensed and transmitted intracellularly through mechanosensors such as p130 CRK-associated proteins, growth factor receptors, or integrin–ECM adhesion plaques [240]. These mechanosensors recruit focal adhesion molecules including FAK, SRC, paxillin, RAC, RHO/RAS GTPases, and Rho-associated kinase to trigger signaling cascades and cytoskeleton organization. These signaling pathways induce changes in cell shape, survival, migration and invasion. The disaggregation of OvCa MCTs, behavior associated with dissemination and metastasis, is promoted by matrix stiffness through mechanotransduction pathways involving ROCK and FAK [35]. YAP/TAZ has been involved with ECM stiffness, cell spreading, proliferation, metastasis, and stem cell-like behavior [109]. Other key mechanosensors involved in mechanotransduction and signaling pathways are the related Hippo pathway as well as MEK/ERK.

Taken together, the mechanical forces in the TME play an important role in cell fate and cancer progression. Understanding the signals involved in mechanotransduction and its correct translation to 3D models, as well as the mechanical characterization of individual cells and on a large scale, are of great importance to improve the modeling of the TME using 3D platforms. Another challenge to overcome would be the heterogeneity in properties found in tumors and their respective cell signaling.

## 4. Conclusions

In this review, we have featured the different 3D systems engineered to model the ovarian TME. Table 1 provides an overview of the highlighted examples and summarizes key advantages and disadvantages. Compared to the 3D systems discussed, designer self-assembling peptides represent an exciting alternative for forming highly organized cell constructs with functional, biochemical and biomechanical features in a user-directed manner. Due to their tunable properties and ability to self-assemble in situ, their integration in microfluidic and cancer-on-a-chip devices would be the next step to refine 3D in vitro models. This will exploit the advantages of both approaches by creating a cell-instructive microenvironment with the desired biochemical and biomechanical properties and facilitating a continuous exchange of the culture medium, metabolites, waste and therapeutic drugs. Additionally, the desired cell populations can be located in different hydrogel formulations in separate chambers within the microfluidic device.

Self-assembling peptides are also increasingly attracting interest as bioinks for 3D bioprinting. They meet key requirements that make them promising candidates for this application, including shear thinning properties and the ability to recover after shear. Commercial products, such as Peptigel^®^, have been used as bioinks to create cell constructs with varying stiffness whilst supporting diverse cell populations [241]. Improved bioinks will advance the use of 3D bioprinting technologies for various biomedical applications and even in cancer research. 3D bioprinting using self-assembling peptides represents an opportunity to create well-defined scaffolds with short fabrication times without compromising cell viability.

We anticipate that the design of 3D OvCa models incorporates not only different cell types but also crucial properties of in vivo tissues, such as the acellular and secreted components of the TME and factors related to tumor spheroid formation, cell–cell and cell–matrix interactions and acquired chemoresistance, to elaborately promote cell proliferation, migration and invasion. In this context, self-assembling peptides hold great promise given the fact that these molecules can be easily designed, having inherent bioactivity and high compatibility to encapsulate cells, integrating essential ECM components, growth factors and key signaling molecules. Overall, the use of self-assembling peptide biomaterials in cancer research will continue to evolve as a function of the advancements in materials science and biology and in our understanding of supramolecular chemistry that facilitates the synthesis of novel materials.

## Figures and Tables

**Table 1:** Three-dimensional models of the ovarian TME.

Model	Characteristics and Advantages	Disadvantages and Limitations	Applications in Cancer Research	Cell Types	Refs.
Mouse models:	Captures in vivo complexity	Ethical concerns	-	-	-
		Costly			
		Time-consuming			
		Special facilities required for housing			
		Requires licenses			
		Murine biology and stroma different from human TME			
Xenografts	Cell lines or patient-derived	Low success rate	Analysis of cancer development and heterogeneity of tumors	HO-8910PM, from patient-derived tissue and ascites	[57,59,61,62,63,64]
	Resemble tumor histology, formation of ascites, gene expression, vasculature, metastatic potential and response to chemotherapy	Possibility of leakage of cancer cells after injection			
	Establishment of tumor biobanks.	Possible downregulation of certain genes and replacement of human stroma by murine stroma	Evaluation of tumor responses to drugs.		
	Resemble patient heterogeneity	Immunodeficient host	Used in parallel with 3D in vitro studies		
Syngeneic	Immunocompetent model	Lack of heterogeneity. Few host strains	Evaluate tumor growth. Model metastasis in peritoneal cavity Study anoikis resistance	ID8	[68,69]
	Rapid growth				
	Easily manipulated				
	Induce metastasis with ascites formation				
	Recapitulate anoikis resistance				
Genetically engineered	Display genetic heterogeneity	Longer time for tumor development. Lack of promoters to develop these models	Model metastasis and cancer progression Study mutation combinations	-	[72,73,74,75]
	Resemble tumor histology				
	Genetically manipulated.				
Laying hen	Display pathological and genetical features similar to patient tumors	Ethical concerns.	Study cancer origin	-	[78,79]
		Lack of native TME			
	Similar developmental pattern to human tumors	Lack of technology-specific for host (e.g., antibodies)			
	High incidence of disease	Lack of protocols			
Spheroids	Resemble cell aggregates found in ascites	Require inclusion of vasculature, immune system components, mechanical signals and fluid dynamics	Study spheroid formation mechanisms.	Ascites-derived cells, SKOV-3, OV-90, OVCAR-3, OVCAR-8, TOV-112, TOV-21, TOV-155	[81,82,83,84,85,86,87,88,89,90,91,92]
	Support different ratios of cancer and stromal cells				
	Mimic nutrient transport, growth kinetics and cell–cell interactions found in solid tumors	Difficulty to image them	Evaluate tumor invasion.		
	Diverse spheroid production techniques	Not all cell lines are capable of forming spheroids			
	Resemble chemoresistance	Different morphology depending on protocol used	Testing of drug delivery systems, drug efficacy and penetration, receptor targeting, cell recruitment abilities and tumor biology.		
	Low cost, ease of use, reproducible, and high-throughput	Lack of native ECM			
Organoids	Maintain histological features	Lack of immune system elements, stromal cells and vasculature.	Study carcinogenesis High-throughput drug screening Genomic analysis	Patient-derived tissue fragments, ascites-derived cells	[93,94,95,96,97,98,99,100,101]
	Mimic genetic features including intra-tumoral				
	High-throughput screening	Costly.			
	Derived from small pieces of tissue	Require supplemental growth factors			
	Can be genetically modified	Intra-tumoral heterogeneity can be lost during passages			
	Creation of biobanks	Mutations are subsequently acquired			
	Maintain cell viability over long periods of time	Need of culture protocols and drug screening strategies			
Microfluidic devices	Commercially available or custom-made devices	Costly	Study tumor development	A2780, TOV112D, OV90, OVCAR5, SKOV-3, ascites-derived cells	[83,102,103,104,105,106,107]
	Include multiple chambers and cell populations				
	Enable fluid perfusion	Special facilities required for manufacture	Resemble cancer dissemination and metastasis		
	Enable formation of spheroids	Predesigned devices cannot be customized			
	Some platforms enable testing pharmcokinetics/dynamics of drugs	Limited recollection of spheroids	Drug screening		
	Variable shear stress	Complex design and use			
	Include nutrient supply and waste removal	Limited material choice	Genomic analysis		
	Maintain cell viability over long periods of time	Lack of cell–cell and cell–matrix interactions			
Natural hydrogels: Matrigel	Contains collagen, laminin, enactin, other ECM molecules and growth factors	Chemically not well-defined	Study tumor biology	SKOV-3, OVCAR-10	[108]
	Cyto-compatible	High batch-to-batch variation			
	Minimally processed	Undefined impurities			
	Mimics in vivo conditions	Limited flexibility to tune the mechanical properties			
	Enables cell–matrix interactions	Quick gelation time			
	Promotes cell growth	Contains growth factors that can cause activation of signaling cascades			
Collagen	Primary constituent of ECM	Batch-to-batch variation	Study tumor biology Evaluate tumor invasion	A2780, OV-NC, OV-206, SKOV-3, OVCAR-3, OvCa433, DOV13, OVSAHO	[38,109,110,111,112]
	Intrinsic cues for cell recognition				
	Similar stiffness to tissues	Limited control over physical and mechanical properties			
	Maintains cell viability over long periods of time	Inability to tailor its composition			
	Enhances cell spheroid and invasion	TME contains different types of collagen and other ECM molecules, not only collagen of a single type			
	Stimulates EMT phenotype	Low mechanical strength			
Synthetic polymer hydrogels (e.g., PEG, GelMA)	Biocompatible	Require cell-binding moieties due to inert nature	Study influence of matrix stiffness on spheroid formation and disease progression	OV-MZ-6, SKOV-3, HO8910, ascites-derived cells.	[113,114,115,116,117,118,119,120,121,122]
	Tunable architecture and stiffness				
	Tailorable with functional ligands	Limited cell recovery			
	Functionalized with ECM proteins or proteolytic degradation sites		Drug screening		
	Enable spheroid formation	Lack of nanofibrous network	Genomic analysis		
	Maintain cell viability over long periods of time		Spheroid formation technique		
Self-assembling peptide hydrogels	Chemically synthesized to enable tunability of properties	Costly	-	-	-
	High design flexibility				
	Reproducible				
	Stable nanofiber network that resembles the ECM				
	Supportive of cell proliferation, invasion and spheroid formation				
PuraMatrix™	Commercially available Low immunogenicity	Poor mechanical strength	Model tumorigenesis and metastasis.	SKOV-3, A2780, A2780/DDP, OVCAR-5.	[123,124,125,126].
			Study influence of matrix stiffness on spheroid formation and disease progression.		
			Drug screening.		
Peptide amphiphiles	Available through custom peptide synthesis.	Low scalability	Study tumor biology	NIH:OVCAR-4.	[127]
	Tailorable with specific signaling motifs				
	Incorporation of ECM proteins		Evaluate influence of matrix stiffness on spheroid formation and disease progression		
	Maintains cell viability over long periods of time	Peptide sequences not normally found in the ECM			
	Supports co-cultures		Drug screening		
	Minimal batch-to batch-variation				

3D, three-dimensional; ECM, extracellular matrix; EMT, epithelial–mesenchymal transition; TME, tumor microenvironment.
